# Implementation of cardiac enhanced recovery after surgery at Lausanne University Hospital, our roadbook to certification

**DOI:** 10.1093/icvts/ivae118

**Published:** 2024-06-17

**Authors:** Zied Ltaief, Mario Verdugo-Marchese, Dan Carel, Ziyad Gunga, Anna Nowacka, Valentine Melly, Valerie Addor, Caroline Botteau, Marius Hennemann, Luc Lavanchy, Matthias Kirsch, Valentina Rancati

**Affiliations:** Department of Intensive Care, Lausanne University Hospital (CHUV), Lausanne, Switzerland; Department of Cardiac Surgery, Lausanne University Hospital (CHUV), Lausanne, Switzerland; Department of Intensive Care, Lausanne University Hospital (CHUV), Lausanne, Switzerland; Department of Cardiac Surgery, Lausanne University Hospital (CHUV), Lausanne, Switzerland; Department of Cardiac Surgery, Lausanne University Hospital (CHUV), Lausanne, Switzerland; Department of Cardiac Surgery, Lausanne University Hospital (CHUV), Lausanne, Switzerland; Department of Development and External Affairs, Lausanne University Hospital (CHUV), Lausanne, Switzerland; Department of Cardio-Respiratory Physiotherapy, Lausanne University Hospital (CHUV), Lausanne, Switzerland; Department of Cardio-Respiratory Physiotherapy, Lausanne University Hospital (CHUV), Lausanne, Switzerland; Department of Anesthesia, Lausanne University Hospital (CHUV), Lausanne, Switzerland; Department of Cardiac Surgery, Lausanne University Hospital (CHUV), Lausanne, Switzerland; Department of Anesthesia, Lausanne University Hospital (CHUV), Lausanne, Switzerland

**Keywords:** Enhanced recovery after surgery, Cardiac surgery, Multidisciplinary team, Patient-centred care, Clinical outcomes, Cost-efficiency

## Abstract

**OBJECTIVES:**

Enhanced recovery after surgery (ERAS) is a multidisciplinary, patient-centred approach aimed at expediting recovery, improving clinical outcomes, and reducing healthcare costs. Initially developed for colorectal surgery, ERAS principles have been successfully applied across various surgical specialties, including cardiac surgery. This study outlines the implementation and certification process of the ERAS program in a tertiary cardiac surgical centre within the Heart-Vessel Department at Lausanne University Hospital.

**METHODS:**

The implementation involved forming a multidisciplinary team, including cardiac surgeons, anaesthesiologists, intensivists, a cardiologist, clinical nurse specialists and physiotherapists. The ERAS nurse coordinator played a central role in organizing meetings, promoting the program, developing protocols, and collecting data. The certification process required adherence to ERAS guidelines, structured training and external evaluation. Key phases included pre-ERAS data collection, protocol dissemination, inclusion of the 1st patients, followed by analysis and full implementation.

**RESULTS:**

Achieving certification required maintaining a compliance rate of over 70% with established protocols. The process involved overcoming various barriers, such as inconsistent practices and the need for multidisciplinary collaboration. In this paper, we provide some solutions to these challenges, including team education, regular meetings and continuous feedback loops. Preliminary data from the initial cohort showed improvements in early mobilization, opioid use, respiratory complications and shorter hospital stays.

**CONCLUSIONS:**

The successful implementation of the ERAS program at our institution demonstrates the feasibility and benefits of a structured, multidisciplinary approach in cardiac surgery. Continuous self-assessment and adherence to guidelines are essential for sustained improvement in patient outcomes and healthcare efficiency.

## Graphical abstract

**Figure:**
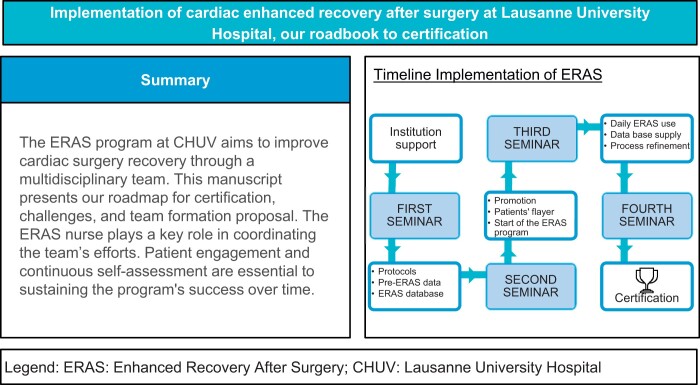

## INTRODUCTION

Enhanced recovery after surgery (ERAS) represents a multidisciplinary, patient-centred care pathway, encompassing the collaborative efforts of surgeons, anaesthesiologists, intensive care physicians, cardiologists, nursing staff, physiotherapists and other pertinent healthcare professionals. This approach is meticulously designed to expedite patient recovery, enhance clinical outcomes and curtail healthcare expenditures. Initially conceptualized for major colorectal surgery [1, 2], the ERAS framework has since been expanded and empirically validated across various surgical disciplines [3]. Notably, in the field of cardiac surgery, the appeal of the ERAS model has grown substantially.

In the early 1990s, Dr Engelman pioneered the fast-track concept in cardiac surgery [4], aiming to facilitate early extubation through minimized opioid usage. The ERAS Cardiac Society emerged in 2017, culminating in the publication of specific guidelines in 2019 [5] in collaboration with the ERAS Society International. Further developments occurred in 2022 when the French Society of Anaesthesia and Intensive Care Medicine, in collaboration with the French Society of Thoracic and Cardiovascular Surgery, released their Guidelines on Enhanced Recovery After Cardiac Surgery [6]. This marked a paradigm shift from traditional, localized care practices to a more structured, integrated approach to patient care.

Recent research [7–9] indicates that implementation of ERAS principles in cardiac surgery could correlate with reduced morbidity and shortened hospital stays. As highlighted by Kamal *et al.* [10], protocols and practices vary greatly, and a lack of standardization clearly affects the robustness of study results. We have chosen in our institution to undertake an initial certification step to obtain the ERAS label, which involves standardizing protocols according to the latest recommendations, a strict external evaluation of compliance with these recommendations, and also the creation of a database including the variables to be studied. In this paper, we delineate our experience with the implementation and certification process of the ERAS program in our tertiary cardiac surgical centre within the Heart-Vessel Department at Lausanne University Hospital.

## METHODS: METHODOLOGY OF IMPLEMENTATION

### Building the team 

#### Cardiac ERAS multidisciplinary team

The inaugural phase of establishing the cardiac ERAS program within our department involved the formation of a multidisciplinary team. Team members were meticulously chosen based on their expertise, experience, dedication and ability to initiate, lead, and advocate for innovative practices. The team consisted of 2 cardiac surgeons, 2 anaesthesiologists, 2 intensivists, a cardiologist, a clinical nurse specialist from the intensive care unit, 2 nurses in cardiac surgery and 2 physiotherapists.

**Figure 1:**
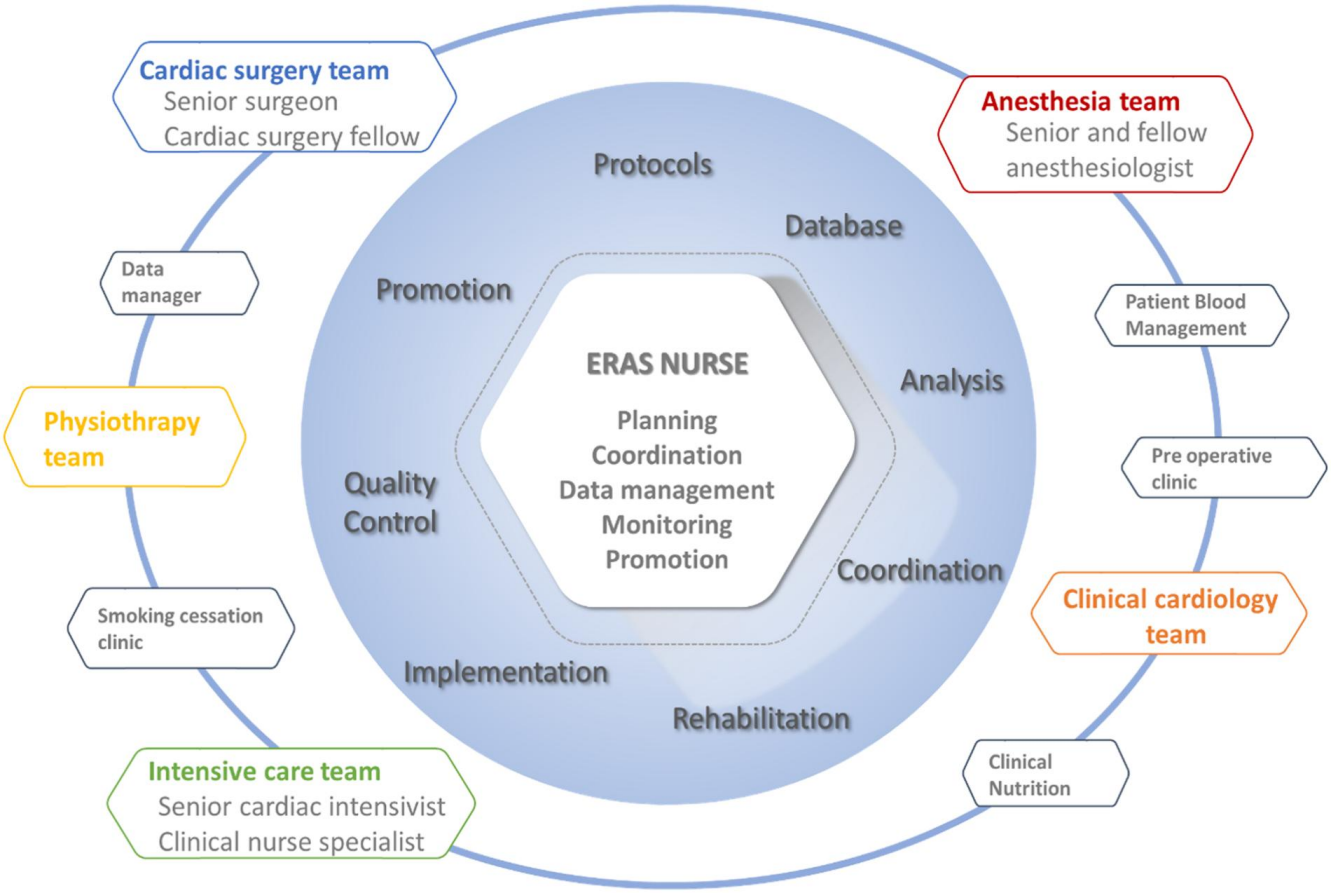
The ERAS multidisciplinary team, which is comprised of various subgroups working together on key implementation tasks, all coordinated by the ERAS nurse. ERAS: enhanced recovery after surgery.

In our cardiac ERAS team, both senior and junior cardiac surgeons play a crucial role in harmonizing surgical practices with ERAS principles. Complementing their efforts, the cardiac anaesthesiologist is instrumental in managing patient blood, optimizing cardiac output and fluid balance, controlling pain and facilitating early extubation. The collaboration between the senior cardiac intensivist and the clinical nurse specialist is vital in postoperative management within the intensive care unit. They lead the charge in early extubation, mobilization and pain management, creating an environment conducive to rapid recovery. The addition of a clinical cardiologist, specialized in postoperative care, is central to coordinating medical and paramedical teams, ensuring seamless care continuity throughout the patient’s journey. Furthermore, physiotherapists are integral members of our team, contributing to the development of preoperative rehabilitation protocols and early postoperative mobilization strategies (Fig. 1).

#### Cardiac ERAS nurse coordinator

**Figure 2:**
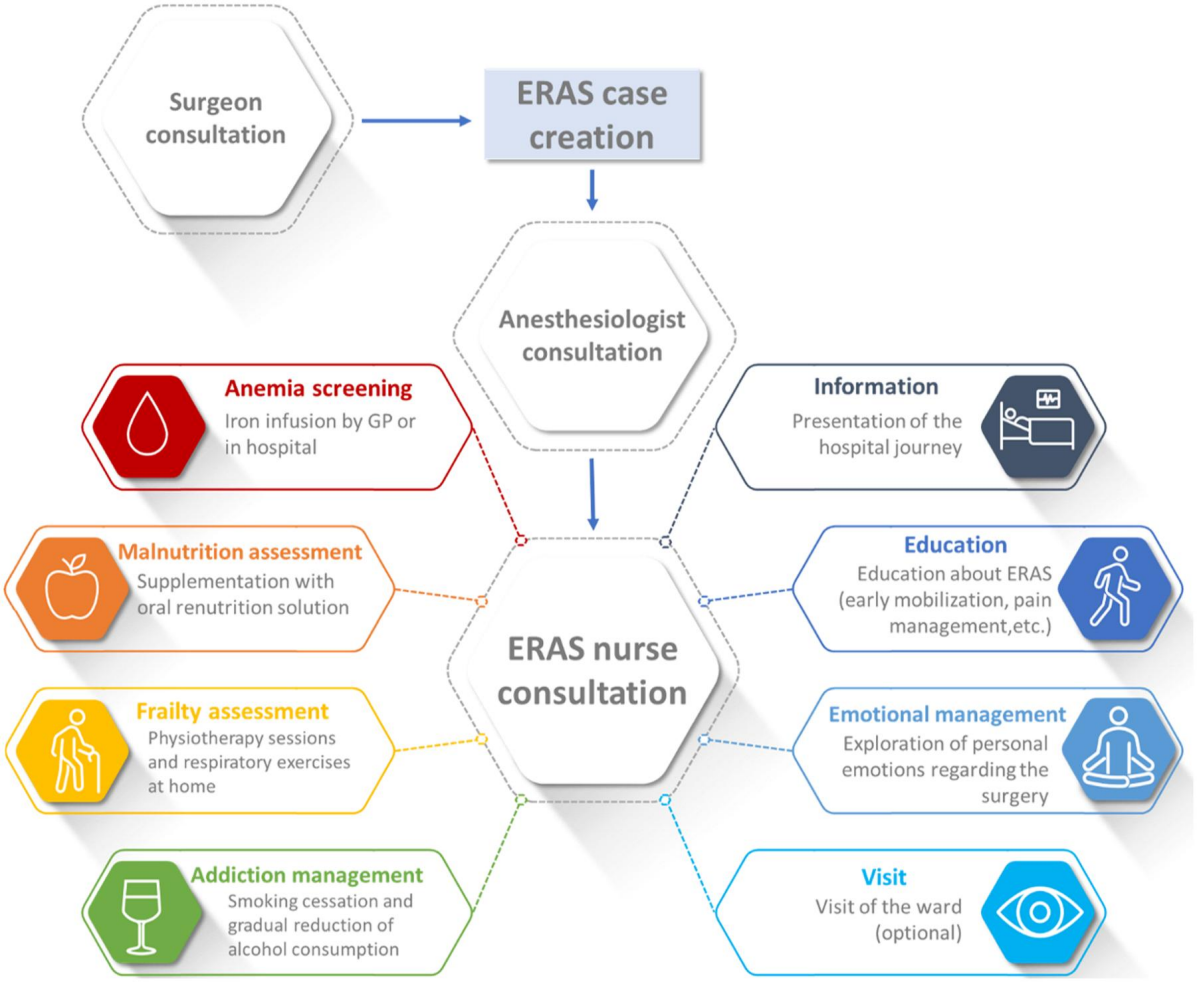
The pre-hospitalization patient pathway leading to ERAS nurse consultation, with the main objectives of the consultation highlighted. ERAS: enhanced recovery after surgery.

The ERAS nurse coordinator assumes a pivotal role in the implementation of the ERAS project within our team, serving as its central organizational hub. Firstly, the coordinator nurse is responsible for organizing meetings to foster timely discussion and collaboration among multidisciplinary team members. In addition, this coordinator plays a pivotal role in tracking the program's progress. Her active promotion of the ERAS program to caregivers was key in developing an environment of awareness, acceptance and adherence to ERAS principles. Moreover, the ERAS nurse coordinator contributes significantly to the development of local protocols, aligning them with existing clinical practices and patient needs and providing critical feedback for protocol updates. Another responsibility is the data collection, a task conducted by 2 ERAS nurses. This data collection is crucial for providing quantitative evaluations of the program’s impact on patient outcomes and healthcare costs, enabling objective assessment and the continual improvement of ERAS protocols. Furthermore, the ERAS nurse was involved in creating patient education materials and coordinating pre-operative rehabilitation sessions with physiotherapists and nutritionists (Fig. 2). During the consultation, the nurse screens the patient for factors such as anaemia, vulnerability, malnutrition, and addiction to alcohol or smoking. In the event of identified issues, the nurse coordinates necessary support, such as anaemia management or sessions with the physiotherapist, dietician or tobaccology consultant. Furthermore, the nurse imparts information to the patient regarding the hospital’s post-cardiac surgery organization by giving all the needed information on ERAS principles, emphasizing early mobilization and pain management. The consultation also provides a platform for patients to express their emotional states concerning the impending surgery. Additionally, the nurse extends an invitation for patients to visit the cardiac surgery ward if they express interest.

Therefore, the role of the ERAS coordination nurse is fundamental to the successful implementation and advancement of the program. The ideal candidate for this role should demonstrate exceptional organizational capabilities, a thorough understanding of both local cardiac surgery practices and ERAS program objectives, and strong interpersonal skills to nurture a positive, collaborative environment among caregivers. We chose a cardiac surgery intermediate care nurse for this position, who brought advanced clinical management expertise, extensive knowledge of digital tools and database management, in addition to the aforementioned skills (Fig. 1).

### The timeline of certification process

**Figure 3:**
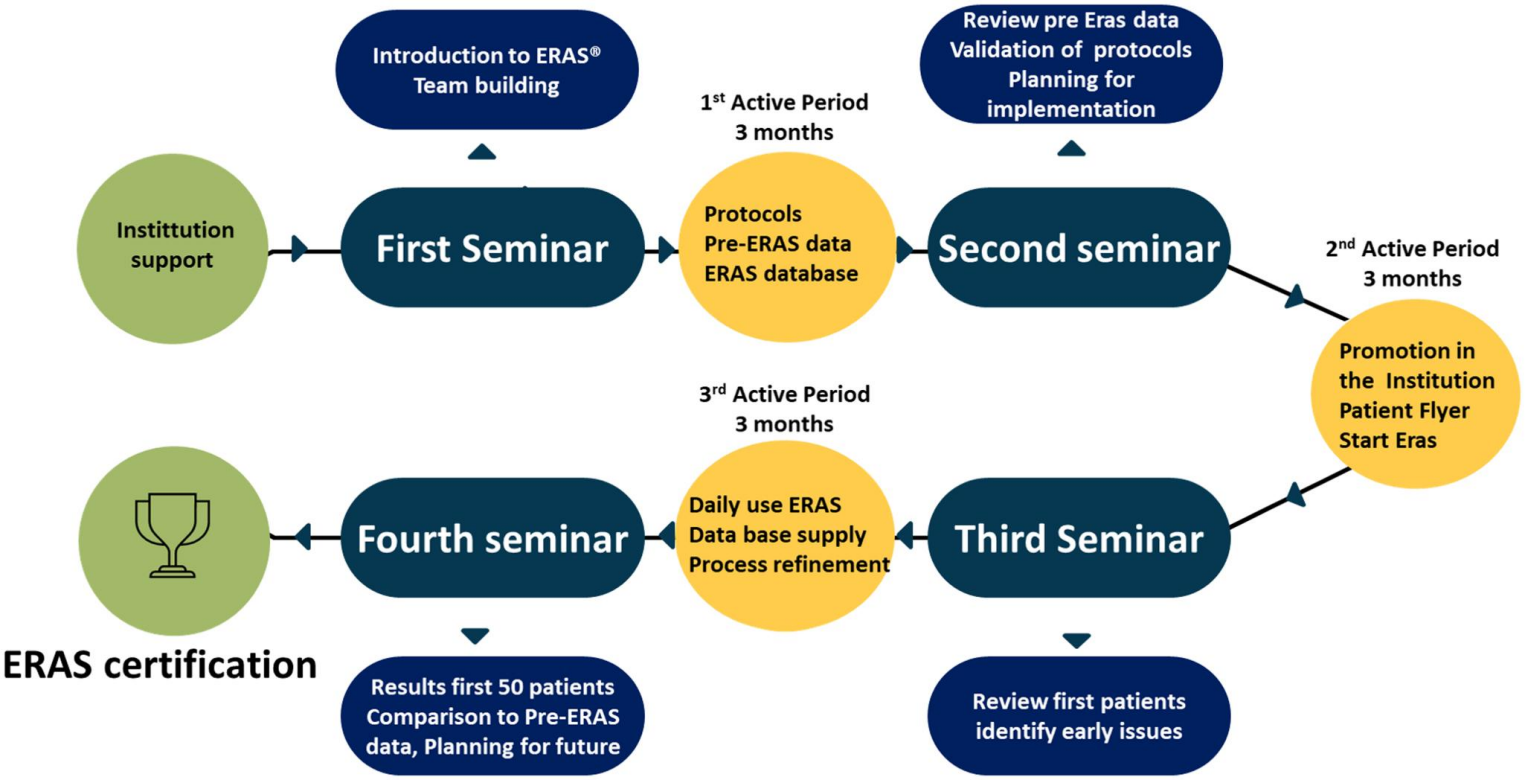
The ERAS certification timeline. ERAS: enhanced recovery after surgery.

The ERAS Society certification process involves establishing a well-functioning ERAS system with clear workflows and protocols accepted by all caregiving staff. Post-implementation, protocol adherence must exceed 70%. The multidisciplinary team must complete a structured training program across 4 seminars over 8 to 10 months, with active implementation phases in between (Fig. 3). An appointed ERAS coach oversees the certification, assessing adherence to and application of principles. Successful completion of this process recognizes the team as an ERAS-Qualified Unit. Further details on each stage are elaborated on subsequent sections of this paper.

#### First seminar: cardiac ERAS team building and education

After the institutional approval, the 1st seminar is held. During this 1st meeting, the ERAS team is formally established, with each member being introduced. The ERAS coach explains the theoretical basis for laying the foundations of the ERAS process, introduces the ERAS system and delineates the global strategy for implementation. As the seminar concludes, the 1st active phase is initiated, with every team member assuming their assigned roles.

##### Active phase 1: pre-ERAS data collection, protocols and database creation

Within the framework of our ERAS program implementation, 3 specialized teams were formed, each tasked with a distinct objective. The 1st team concentrated on gathering pre-ERAS data from the last 50 consecutive cardiac surgery patients in our institution. The 2nd team was assigned the task of tailoring the general Cardiac ERAS guidelines to fit our institutional practices, drafting local protocols while remaining true to the essence of ERAS. The 3rd team embarked on developing an institutional database specifically for cardiac surgery ERAS patients. In line with ERAS recommendations, weekly meetings were scheduled to deliberate on all protocols and achieve consensus.

During the implementation and evolution of our ERAS program, we incorporated a close multidisciplinary collaboration with various hospital departments. This collaboration included partnerships with Patient Blood Management team, Clinical Nutrition, Physiotherapy and the Smoking Cessation Clinic, among others (Fig. 1).

#### Second seminar: analysing pre-ERAS outcomes, validating protocols and implementing strategies

During the 2nd seminar, a thorough review and analysis of the pre-ERAS data from 50 patients were carried out. We observed mixed adherence to guidelines. For instance, we achieved early extubation in 88% of cases, multimodal analgesia in 74%, and both antibiotic prophylaxis and tranexamic acid use in over 96% of patients. However, we also identified significant gaps in our practices. Preoperative anaemia management was inconsistent, and we lacked formal strategies for detecting and managing frailty and undernutrition, as well as for smoking and alcohol cessation. Atrial fibrillation prophylaxis was notably low, implemented in only 2% of patients. Additionally, there was no practice of nasal decontamination, and early mobilization was achieved in only 8% of cases, with a high level of postoperative opioid use persisting, as more than 50% of patients remained on opioids by day 3 with a high level of overall respiratory complications (36%), ileus (52%) and high hospital stay (near 10 days). Subsequently, we engaged in a thorough discussion to align our practices more closely with both our pre-ERAS results and the ERAS Society guidelines, deciding on which protocols needed further implementation or revision. Finally, we decided which category of patients to start with at the beginning (planned valvular surgery), and we fixed a date to kick-off the inclusion of the 1st cardiac ERAS patient.

##### Active phase 2: protocol dissemination and inclusion of the 1st patients

During this crucial phase, each team member conducted informative sessions within their respective departments. The primary objectives of these sessions were to communicate the changes introduced by the ERAS program, elucidate the anticipated benefits and, most importantly, support colleagues in enhancing their abilities to implement best practice recommendations in the standard of daily care, backed by the leadership experts. Another key goal during this period was to enrol a minimum of 20 patients in the program and systematically input their data into our newly established database. All team members were also tasked with identifying and recording any challenges encountered (some examples are provided in Table 1), which were then deliberated in our weekly meetings.

**Table 1:** Examples of challenges and solutions in our experience implementing ERAS in cardiac surgery

Barriers	Description	Solution
Short delay between consultation and surgery	Short delay to prepare patients for ERAS between surgical consultation and the day of surgery.	Same day consultations: Patients are seen the same day by ERAS nurse and anaesthesiologist to expedite preparation.
Patient compliance	Some patients are reluctant to participate in early mobilization and other ERAS protocols.	Education and engagement: Provide thorough education to patients well before surgery, supply them with brochures about the ERAS program, and give them a journal to fill out during their stay to actively participate in the program.
Which protocols	Selecting which new protocols to implement.	Feasibility assessment: Decide collectively to implement only feasible new protocols that can be realistically adopted and integrated into the existing system.
Diffusion of information and new protocols	Ensuring effective dissemination of new protocols and information.	Team meetings and intranet: Conduct regular team meetings in each department and use the intranet to distribute protocols and information widely.
Frailty screening, tobacco cessation, malnutrition screening	Ensuring comprehensive screening for frailty, tobacco use, and malnutrition.	ERAS nurse consultations: Utilize ERAS nurse consultations to perform thorough screenings and interventions.
Timing and structure for anaemia screening and IV iron administration	Coordinating the administration of IV iron in a timely manner.	General practitioner network: Engage general practitioners to facilitate timely anaemia screening and administration of IV iron.
Practitioner reluctance to same day mobilization	Addressing the reluctance of practitioners to implement same-day mobilization.	Specialist ICU nurses: Deploy ICU nurse specialists to assist with initial mobilization and maintain existing protocols and contraindications for mobilization.

ERAS: enhanced recovery after surgery; ICU: intensive care unit; IV: intravenous.

A notable accomplishment in this phase was the successful pilot of the ERAS consultation. This initiative was structured to utilize the combined expertise of our nurses, physiotherapist, nutritionist and anaesthesiologist, representing a significant step in integrating multidisciplinary care into the preoperative pathway (Fig. 2).

## PRELIMINARY RESULTS AND CERTIFICATION

### Third seminar: analysing the 1st results

In this meeting, we analysed data from the initial cohort of 20 patients enrolled in the program. Under the guidance of the ERAS coach, we scrutinized the primary outcome measures. This analysis facilitated the identification of potential areas for improvement. Additionally, we assessed the compliance statistics with ERAS protocols, highlighting any points of conflict. During this stage, the ERAS trainer provided invaluable insights, drawing on experiences from other specialties and institutions to offer practical suggestions for enhancement. Team members also had the opportunity to share their experiences, particularly focusing on the challenges they faced in terms of organization and communication.

#### Active phase 3: full implementation

All patients scheduled for cardiac surgery were included in the ERAS program. To ensure the continuous improvement of the protocol, bimonthly meetings were maintained. A significant challenge during this phase was maintaining high compliance rates while accommodating the increasing number of patients. This period proved to be critical, and the persistent involvement of ERAS team members in clinical settings was a key factor in its success and in assisting caregivers to adhere to the protocols effectively.

### Final seminar: compliance calculation, outcome results and ERAS certification

To attain certification, ERAS teams must demonstrate a minimum 70% compliance rate with the protocols. The primary objective of the concluding meeting was to conduct a thorough analysis of data from the first 50 patients enrolled in the ERAS program, focusing on 2 key aspects. The 1st aspect involved evaluating adherence to the protocols, particularly after the program had stabilized operationally. For example, we reached a compliance of 78% in atrial fibrillation prophylaxis, 71% in nasal decontamination, 50% early mobilization (which represents 86% of patients who met all criteria for mobilization) and 96% of patients were screened for anaemia, frailty and undernutrition. The 2nd aspect aimed to assess the impact of the protocol on patient outcomes, achieved by comparing data from the ERAS patients with that of an historical cohort, and identifying any potential areas for improvement. Preliminary data from the accreditation process have shown clinically significant improvements in early mobilization (42% improvement at postoperative day 1), opioid use (23% reduction at postoperative day 3), lower respiratory complications (19% reduction), 36 hours earlier time to bowel function recovery and a decrease in postoperative delirium. Data from our prospective cohort and the ongoing registry are expected to provide insights that better identify the potential impact of our program on patient outcomes and hospital costs. Following these analyses, team members provided valuable feedback, sharing experiences from each implementation stage. These insights were particularly beneficial for the ERAS trainer in planning future implementations.

## DISCUSSION

In our journey to implement the ERAS program within our cardiac surgery department, we encountered a spectrum of challenges that tested our resilience and adaptability.

### Institutional challenges

As a leading pioneer in Switzerland and Europe for the ERAS program, our hospital initiated its 1st program in Visceral Surgery in 2011. This was followed by implementations in urology, thoracic surgery and, more recently, neurosurgery [11–14]. These adaptations consistently resulted in reduced postoperative complications, shorter hospital stays [15], improved patient satisfaction and lowered healthcare costs [16, 17]; this has been demonstrated in several studies across different types of surgeries [18, 19]. Recognizing the program’s extensive potential, efforts were made to encourage participation across more services and specialties. Our institution is well aware of the potential of such a program, and an initiative to encourage latecomer services and specialties was taken. The resource-consuming nature of ERAS implementation, including staff training, patient education and continuous data monitoring for quality improvement, poses significant logistical and financial challenges. The institutional commitment is the foundation stone essential to any team about to start its ERAS program, providing the necessary human and material resources needed to create, implement, and maintain the program.

### Specific challenges related to cardiac surgery

The implementation of the ERAS program in the sphere of cardiac surgery presents distinct challenges. Achieving haemodynamic stability, managing bleeding complications, the necessity for prolonged mechanical ventilation and intensive care unit stay in some cases pose hurdles to early mobilization and oral nutrition, thereby delaying the activation of postoperative ERAS protocols. Therefore, the synchronization of ERAS principles with established intensive care protocols demands a meticulous, collaborative approach to ensure patient safety while striving for accelerated recovery in the postoperative period.

### The patient as a key player in the process

Patient engagement is a crucial element of the ERAS program in cardiac care, playing a significant role in ensuring optimal outcomes. Active participation of patients in their care pathway not only deepens their understanding of the perioperative process but also promotes adherence to ERAS protocols. In the preoperative phase, the nurse coordinator details the ERAS program and outlines the perioperative course. This includes educating patients about their upcoming surgical procedures, postoperative expectations, potential complications and recovery benchmarks. During these consultations, patients are encouraged to adopt lifestyle modifications, such as quitting smoking and limiting alcohol consumption, which can markedly improve their perioperative experience.

In the postoperative phase, patients receive booklets to monitor their progress and record any concerns or questions. This approach empowers patients, enhancing their autonomy and engagement in the care process, and fosters a collaborative atmosphere with healthcare professionals. This method represents a shift from a traditional, paternalistic model of care to one that positions the patient as an active participant in their treatment.

Moreover, patient involvement in their own recovery journey is instrumental in the success of the ERAS program, highlighting the importance of patient-centred care in contemporary medical practices. Upon discharge, patients are invited to complete a structured institutional questionnaire. This tool serves as an objective method to assess the impact of the ERAS program on the patient’s perioperative experience.

### Program financial challenge

Human resources are the primary expense during the ERAS program’s development and implementation. Initially, 3 physicians dedicated at least 5% of their total work time to drafting objectives and protocols. A nurse coordinator role was established at 40% time, with an additional nurse at 30% time during the 1st year. A part-time data manager was also appointed, dedicating a minimum of 5% of their time to database development. The development phase included 4 multidisciplinary meetings and 3 focused on database development. During the implementation and certification phase, 4 seminars were conducted with the ERAS society coach, with bimonthly meetings totalling 15 by the ERAS team members.

These upfront costs are expected to be offset by the ERAS program's ability to significantly reduce rates of complications, intensive care usage, and hospital stays. Ultimately, these improvements not only enhance patient outcomes but also position the ERAS program as a cost-saving initiative, making it a financially viable option for healthcare institutions.

### After-certification challenges

In cardiac surgery centres certified by ERAS Society, achieving certification marks the beginning of a sustained commitment to excellence and continuous evaluation. Foremost, ongoing self-assessment is critical. There must be consistent adherence to the established ERAS guidelines, with immediate identification and rectification of any deviations, to preserve the program’s integrity and effectiveness. Secondly, empirical evaluation of the program's impact is essential. Conducting detailed studies to compare postoperative complications before and after the program’s initiation provides tangible evidence of its effectiveness and highlights areas for improvement.

## CONCLUSION

The adoption of ERAS in cardiac surgery represents a significant shift towards a comprehensive, patient-centred approach. In our institution, the successful implementation of this model has been largely attributable to the effective collaboration of a multidisciplinary team. Each member plays an integral role in enhancing patient outcomes, with the coordinator nurse being pivotal in ensuring fluid coordination and communication across various specialties. Our commitment to a patient-centred approach and adherence to the latest ERAS guidelines culminated in achieving ERAS label certification, distinguishing us as the 1st centre in Europe to do so. This achievement not only reinforces our resolve to improve patient satisfaction, reduce postoperative complications and expedite recovery times but also serves as a catalyst for ongoing improvement.

However, the essence of ERAS lies in its capacity for adaptability. Continuous self-assessment allows healthcare professionals to evaluate the program’s effectiveness, pinpoint areas needing enhancement and refine their strategies. This dynamic process ensures that ERAS protocols remain at the forefront of surgical care. Looking ahead, our challenge in the coming years will be to maintain this momentum of continuous evolution and improvement in the ERAS program.

## Data Availability

Data are available on request.

## ABBREVIATION

ERAS: Enhanced recovery after surgery
